# Impact of L-PRF on pain and healing outcomes in lower third molar surgery: a randomized split-mouth trial

**DOI:** 10.1590/1807-3107bor-2024.vol38.0089

**Published:** 2024-09-13

**Authors:** Raissa Pinheiro MORAES, Fábio Wildson Gurgel COSTA, Paulo Goberlânio de Barros SILVA, Francisco Samuel Rodrigues CARVALHO, Jéssica Emanuella Rocha Moura PAZ, Gabriel Carvalho MATOS, Marcela Lima GURGEL, Edson Luiz CETIRA FILHO, Eduardo Costa Studart SOARES

**Affiliations:** (a) Universidade Federal do Ceará – UFCE, Postgraduate Program in Dentistry, Fortaleza, CE, Brazil.; (b) Universidade de São Paulo – USP, School of Dentistry of Ribeirao Preto, Ribeirão Preto, SP, Brazil.; (c) Private Dental Office, Fortaleza, CE, Brazil.; (d) Universidade Federal do Ceará – UFCE, Postgraduate Program in Dentistry, Fortaleza, CE, Brazil.

**Keywords:** Biological Products, Pain, Wound Healing, Oral Surgical Procedures, Tooth Socket

## Abstract

This study explored the effects of L-PRF on pain, soft tissue healing, periodontal condition, and post-extraction bone repair of mandibular third molars (3Ms). A randomized, prospective, triple-blind, split-mouth clinical trial was conducted with 34 volunteers. Eligible patients were randomly allocated into two treatments: G1 (without L-PRF), G2 (alveoli filled with L-PRF), in which the removal of bilateral 3Ms was performed at the same surgical time. Outcomes were assessed according to a visual analogue scale (pain), soft tissue scoring system (wound healing), periodontal probing of mandibular second molar. Bone repair was determined by volumetric analysis (ITK-SNAP software) and fractal analysis (ImageJ software). An intention-to-treat approach to Statistical analysis was used. L-PRF reduced pain in the 7-day postoperative follow-up (p = 0.019) and not only improved soft tissue healing after 1 month of follow-up (p = 0.021), but also probing depth (distal face) in 3 months postoperatively (p = 0.011). Significant alveolar reduction occurred in 3 months after surgery in both treatments (p < 0.05), however, this was more significant in G1 (p = 0.016). The fractal dimension showed no statistical differences. L-PRF improved postoperative clinical parameters of pain, soft tissue healing, and periodontal condition, suggesting that it has a beneficial effect on preserving the alveolar ridge and accelerating the initial repair process.

## Introduction

Mandibular third molar surgery is a procedure frequently associated with postoperative discomfort, primarily due to the inflammatory events that naturally occur because of the surgical trauma.^1^ Therefore, it is essential to closely monitor the healing process and bone remodeling to prevent unfavorable outcomes.^2^

Numerous studies have been conducted with a focus on strategies performed with the aim of not only reducing postoperative symptoms but also enhancing and accelerating the healing process. For this purpose, biomaterials such as platelet aggregates have been developed. These aggregates have the potential to regulate inflammatory events and promote tissue regeneration, thereby contributing to the treatment of bone defects.^3^

Platelet aggregates can be categorized into various types, with two notable variants: Platelet-Rich Plasma (PRP) and Leukocyte-Platelet-Rich Fibrin (L-PRF). Distinct protocols are employed to obtain each type of platelet, involving differences in centrifugation protocol, impact on injured tissue, and cellular composition, among other factors.^4^

L-PRF, a natural alternative to PRP, is gaining popularity in maxillofacial surgery because of its biological advantages. These benefits arise from its denser fibrin network structure, gradual release of growth factors and cytokines, presence of mononuclear leukocytes, integration of circulating microparticles and adhesive glycoproteins, and its easy, quick, and cost-effective processing.^5^ Moreover, another possible benefit of L-PRF on third molar alveoli is related to the periodontal health of second molars. Surgical removal of fully impacted third molars has been found to lead to loss of attachment on the distal portion of the second molar, irrespective of the flap design used or the initial height of the alveolar bone.^6^ A longitudinal study has shown that third molar extraction leads to periodontal breakdown on the distal surface of the second molar, leading to increased probing depth, and immunological-microbiological changes.^7^

The widespread adoption of L-PRF for alveolar filling after mandibular third molar extraction is due to its numerous advantages. However, its impact on postoperative morbidity remains unclear. A recent meta-analysis had the aim of assessing the effects of novel PRF centrifugation methods on postoperative outcomes and soft tissue healing after mandibular third molar removal. This meta-analysis took into account that surgery of mandibular teeth involves more invasive procedures, such as larger flaps, ostectomies, and odontosections. These types of procedures generally generate greater postoperative morbidity The aforementioned analysis found a limited number of studies that specifically examined the benefits of L-PRF using a split-mouth design, with only four trials meeting the inclusion criteria.^6^ Moreover, only one study in the meta-analysis addressed bone healing as an outcome.^7^ This emphasizes a significant gap in the current literature relative to the application of L-PRF in mandibular third molar surgery.

The lack of studies on this specific topic highlights the need for additional research to fully comprehend the potential benefits and limitations of L-PRF in this context of promoting favorable outcomes and enhancing bone healing. In view of the foregoing, the aim of our present study was to evaluate whether L-PRF has positive effects on pain, soft tissue healing, periodontal condition, and, most importantly, the quantity and quality of bone repair after mandibular third molar extraction.

## Methods

### Trial design

A triple-blind, crossover, clinical trial was conducted at the Walter Cantídio University Hospital, Federal University of Ceará, from November 2019 to December 2020. Patients referred for bilateral mandibular third molar extraction participate in the studs, with approval from the Research Ethics Committee (CAAE 19487419.0.0000.5054). A term of Informed consent was obtained in accordance with the Declaration of Helsinki, and the study followed the CONSORT guidelines for quality and transparency.^8^

### Sample size and power

Two sample size calculations were conducted, one for bone repair and another for clinical variables, with the larger value chosen. Following the research of Ritto et al.,^9^ which showed a significant increase in bone mineral density with L-PRF (954.100±500.768 vs. 522.514±352.281), 23 patients were initially estimated to meet the study requirements relative to statistical power and confidence level (90% power, 95% confidence). To account for potential dropouts, an additional 20% was added, resulting in a total of 28 patients. As the study involved bilateral mandibular third molar extraction (split-mouth) from each patient, a total of 56 extractions were performed in all 28 participants.

With a grade 5 healing score of 96.4% for the L-PRF group compared with 67.9% for the non-L-PRF group, the present sample size of 28 patients per treatment yielded a statistical power of 81.29% to reject the null hypothesis. In a new sample size calculation, a total of 28 patients was necessary for a power of 80%.

### Eligibility criteria

This study sample consisted of individuals aged 18 to 35 years, classified as healthy according to the American Society of Anesthesiologists (ASA I). Participants included both males and females with bilaterally located mandibular second and third molars. Furthermore, in the thorough clinical examination, they showed no signs of inflammation in the periodontal tissues and were devoid of pain, local inflammation, or associated pathologies. The criteria for inclusion of third molars entailed consistent bilateral patterns of root formation, positioning, and level of impaction, in accordance with the classification established by Pell, Gregory, and Winter. Patients included in the study were required to have previously been recommended for the extraction of their mandibular third molars, and their willingness to participate was confirmed by their informed consent, a term that was read and signed prior to their involvement.

Exclusion criteria referred to pregnant women, smokers, individuals with systemic conditions that could affect inflammation and healing reactions, and those with a platelet count below 150,000 ml/mm3. Furthermore, participants who missed postoperative assessments, did not follow recommendations, experienced intraoperative complications, or had surgical procedures exceeding a 15-minute interval between each side (measured from starting the incision through to removal of tooth) were also excluded from the study.

### Treatment characterization and randomization

The patients initially underwent evaluation by the lead researcher. This consisted of a combination of medical history and clinical examination. The data collected were recorded on a standardized patient chart specifically designed for the study. This information included details about the chief complaint, dental and medical history, degree of tooth impaction (classified radiographically by using Pell and Gregory [1933] and Winter [1929] criteria), and stages of development of the tooth and root (Nolla stages). Data from the Pernambuco Index (PI)^11^ were also included. These were used to assess surgical complexity based on factors such as occlusal plane level, retromolar space, tooth angulation, curvature and root count, relationship with the second molar, patient’s age, and Body Mass Index (BMI). During postoperative follow-up time intervals, a calibrated examiner measured all the variables being analyzed.

Participants were randomly allocated to two treatment groups: G1 (control), which involved extraction without L-PRF insertion, and G2 (experimental intervention), where L-PRF was inserted into the alveolar socket during extraction. To maintain patient blinding relative to the alveolus receiving L-PRF treatment, surgical drapes were used to cover patients’ eyes, and the platelet aggregate was discreetly introduced Into the alveolar socket

Each participant had both mandibular third molars extracted during a single surgical session. The allocation of treatments to specific alveolar sockets was randomized using the “randbetween” function in Microsoft Excel® software, version 2010. Random numbers were generated and sealed in opaque envelopes, which were held by a collaborator not involved in surgical procedures or outcome assessments. The envelopes were opened only after the removal of both mandibular teeth. The side for starting the surgery was also randomly determined.

### Surgical procedure

To ensure the safety of both patients and researchers, stringent biosafety protocols were followed. This included the use of sterile instruments, hydrogen peroxide mouthwash, and the wearing of appropriate personal protective equipment. As a proactive measure to mitigate the potential spread of the COVID-19 (SARS-CoV-2) pandemic, scheduling was adjusted to accommodate only one patient per shift, and the clinic was exclusively reserved for the designated surgical procedure. Furthermore, strict measures were implemented to prevent individuals with COVID-19 symptoms from visiting the clinic.

All surgical procedures were performed by a single surgeon in the same outpatient clinic, in accordance with standardized techniques for third molar extraction. The procedure began with the administration of local anesthesia, using 4% articaine hydrochloride with 1:200,000 adrenaline (Articaine®, DFL, Rio de Janeiro, Brazil). Subsequent to anesthesia, a triangular flap was created, and any bone obstructing tooth extraction was removed using a high-speed handpiece with a proficient carbide bur (#702, FGXL, Brazil), continually irrigated with a 0.9% saline solution. Tooth sectioning with the #702 bur and subsequent removal were performed, if necessary, using either a straight or Seldin elevator. All operative steps were performed consistently on both sides.

### Platelet preparation

From each patient, 20 ml of venous blood was collected and immediately transferred to sterile glass tubes with no anticoagulants or interfering substances. These tubes were then centrifuged using a Montserrat centrifuge (Oxford, England) in accordance with Choukroun’s protocol (3,000 rpm for 10 minutes), a method widely used in the literature.^12^ This process resulted in a product with acellular plasma at the top, L-PRF gel in the middle, and red blood cells at the bottom. The L-PRF gel was meticulously extracted from the glass tube using sterile instruments, separating it from other residual blood components.

After tooth extraction, the operating surgeon (researcher) left the operating room. Then, a second surgeon and an assistant entered the room to insert the L-PRF into the surgical flap on one side and suture both surgical sites. This approach ensured that the researcher remained unaware of the insertion procedure, maintaining blindness relative to assignment of the treatments to each side. Suturing was performed using 4-0 nylon threads. After suturing, the patient was instructed to bite down on a piece of gauze for 30 minutes and to follow the postoperative guidelines provided.

### Drug protocol

Patients were instructed to adhere to a medication regimen that included taking amoxicillin 500 mg every eight hours for seven days. They were also advised to take ibuprofen 600 mg every twelve hours for three days. Moreover, dipyrone was prescribed as an additional analgesic option, with a recommended dosage of 500 mg every six hours for three days.

### Pain and soft tissue assessments

Postoperative pain was assessed at twelve-hour intervals and one week after the procedure using the Visual Analog Scale (VAS), with separate scales for each side.^9^ The healing was evaluated on the seventh day. and one month after the operation. For this assessment, an adapted version of the healing index (HI) scores, developed by Landry and colleagues, was used.^13^ This score provides a comprehensive evaluation of the tissue, considering factors such as incision margin epithelialization, tissue color, response to palpation (presence or absence of bleeding), presence of granulation tissue, and incidence of suppuration.^14^

### Periodontal condition

Moreover, before surgery and three months after surgery, the probing depth on the distal (D), distobuccal (DB), and distolingual (DL) sites of the mandibular second molar was measured using a North Carolina probe (no. 15). The probe was inserted into the gingival sulcus along the longitudinal axis of the tooth until slight resistance was encountered.

### Imaging evaluation

Cone-beam computed tomography (CBCT) scans were performed using a Kodak K 9000 3D machine (Kodak Dental Systems, Carestream Health, Toronto, Canada). The scans were performed with a kilovoltage range of 65 to 75 Kvp, using 8 to 12 mA, and an exposure time of 23 seconds. These CBCT images were obtained at time intervals immediately after the surgical procedure (T0), as well as at one month (T1) and three months (T2) after the surgery. Importantly, the images were stored in Digital Imaging and Communications in Medicine (DICOM) format, enabling subsequent assessment of alveolar bone volume and quality.

The alveolar bone volume and quality were evaluated by an examiner who was unaware of the particular surgical protocol used, ensuring a blinded evaluation process.

### Volumetric analysis

Alveolar volumes were calculated at two specific time points by an experienced evaluator (M.L.G.) who was skilled in performing three-dimensional segmentation and image processing: immediately after the surgical procedure (T0) and three months post-surgery (T2). This was accomplished using ITK-SNAP software, version 3.8 (http://www.itksnap.org/), which allowed for the processing of tomographic images from both the right and left alveoli at both stages.

**Figure 1:**
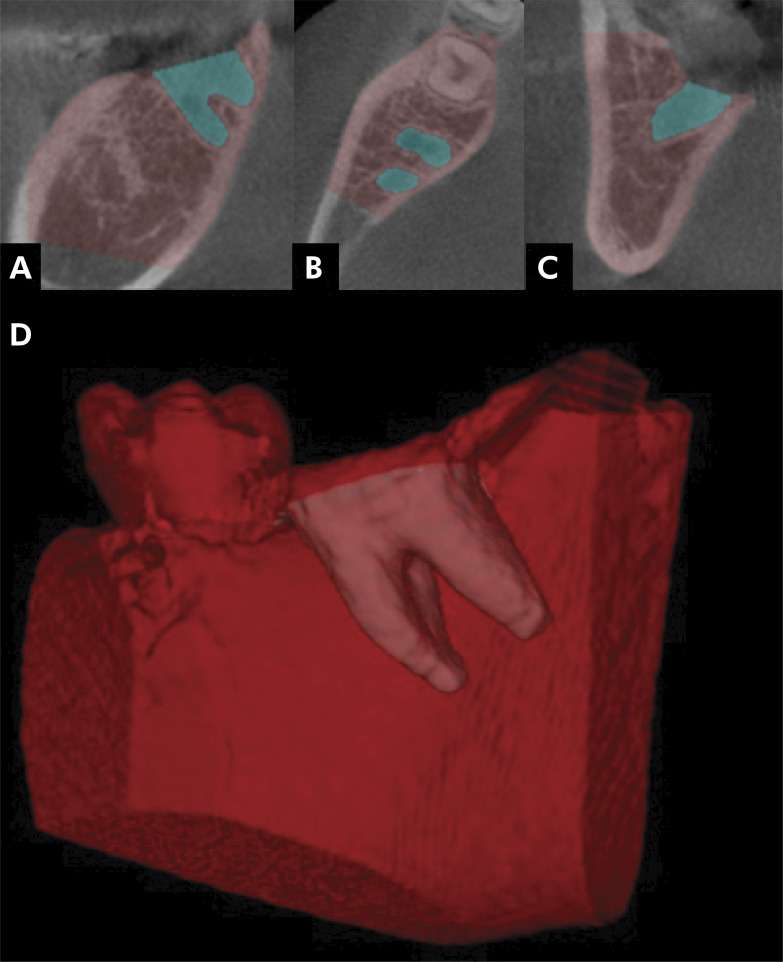
Alveolar reconstruction using the ITK-SNAP software. A. Sagittal slices; B. Axial slice; C. Coronal slice; D. Three-dimensional reconstruction of alveoli for volume calculation.

The process started with conversion of the DICOM images into the Neuroimaging Informatics Technology Initiative (NIfTI) format. After this, regions of interest (ROIs) were segmented to cover the entire length of the alveoli. The software then automatically calculated the alveolar volumes for these segmented regions, provided measurements in cubic millimeters (cm^3^) (Figure 1).

### Fractal analysis

The bone quality was also assessed in a blinded manner by a calibrated researcher (R.M.P.), This involved fractal analysis (FA) at three time intervals: immediately after the surgical procedure (T0), one-month post-surgery (T1), and three months post-surgery (T2). The process began by selecting coronal sections from the original tomographic images. These sections were chosen at various thicknesses: PR1 (panoramic radiograph with a 1 mm thickness), PR5 (panoramic radiograph with a 5 mm thickness), and PR10 (panoramic radiograph with a 10 mm thickness). The mean values of these sections were then calculated and represented as the mean PR value. Furthermore, a sagittal section labeled as SR1 (sagittal radiograph with a thickness of 1 mm) was chosen.

The scans chosen were exported as JPEG (Joint Photographic Experts Group) images to the Image J software (https://imagej.nih.gov/). The process of calculating the fractal dimension occurred after defining a region of interest (ROI) that covered the extent of surgical alveolus, while excluding the crestal lamina dura. To assess the images, the box-counting method introduced by White and Rudolph^15^ was used. These images were saved with acquisition of grayscale images with a bit depth of 8.

For each CT scan (T0, T1, and T2) of each patient, an internal control measurement of 5x5 mm was defined, located consistently in an area shared across all scans. This region was typically situated between the mandibular foramen and the base of the mandible. This internal control was used to compare the FA values in the sagittal slices. The ROI for this control was a spherical measurement of 5x5 mm.

**Figure 2:**
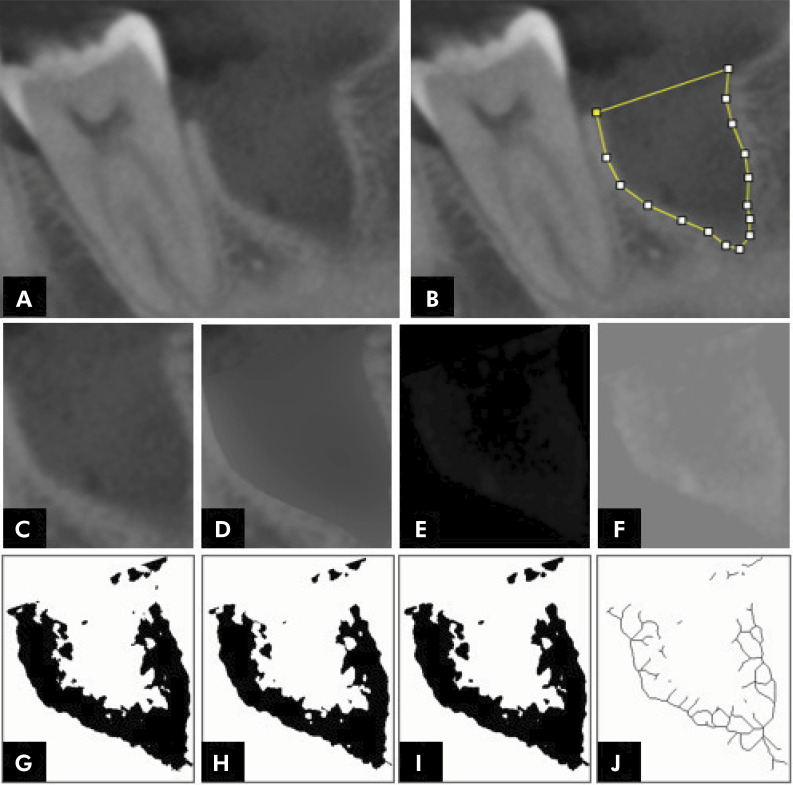
Step-by-step analysis of the fractal dimension of the dental alveoli. A. Coronal slice (5-mm thick) of a CBCT scan; B. Delimitation of the region of interest (ROI) C. ROI (dental socket); D. Blurred image with Gaussian filter; E. Image subtraction result; F. – Addition of a gray value threshold of 128; G. Binary image; H. Eroded image; I. Dilated image; J. Skeletonized image.

The subsequent steps in the FA calculation included duplicating the chosen ROI image (excluding the crestal lamina dura), applying a Gaussian filter (sigma-35), subtracting it from the original image, adding a gray value of 128, binarizing the image, and then subjecting it to erosion, dilation, and skeletonization. These processes were performed to obtain the fractal dimension value (Figure 2).

### Statistical analysis

Quantitative data were presented in terms of mean and standard deviation values and subjected to the Kolmogorov-Smirnov normality test. Subsequently, pairwise comparisons were made using either the Wilcoxon or Friedman/Dunn tests. Whereas categorical data were represented as absolute and percentage frequency values, and they were compared by using the McNemar test.

All analyses were performed by adhering to a 95% confidence interval, the difference limit defined as significant between groups was p < 0.05, and the procedures were performed within the SPSS® (Statistical Package for the Social Sciences) version 20.0 software designed for Windows®. An intention-to-treat (ITT) approach was used, incorporating all randomized patients who were included in the study. Moreover, a treatment received analysis was performed, focusing on patients who actually underwent the intervention, thereby assessing the effectiveness and outcomes of the treatment within the intended population.

## RESULTS

### Sample characterization

**Figure 3:**
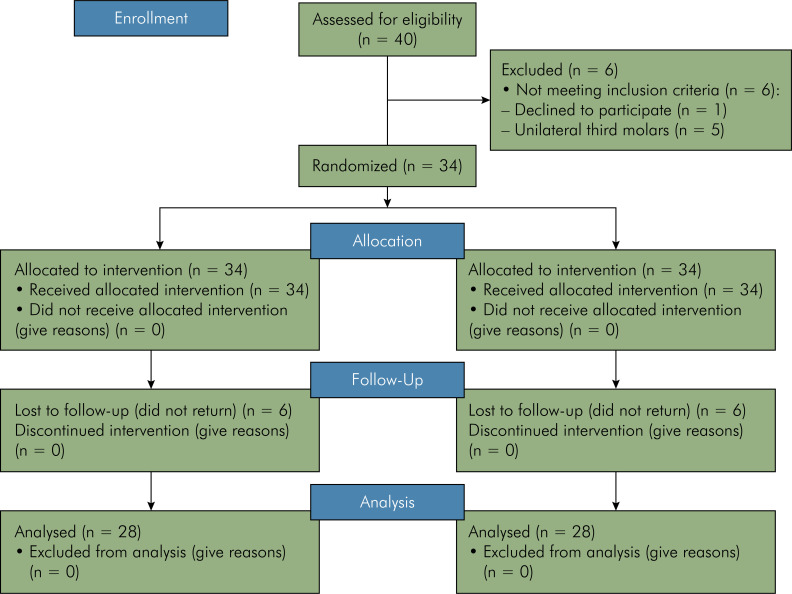
Flow Diagram Following the CONSORT Statement.

A total of forty patients were initially assessed, but six were excluded because they did not meet the inclusion criteria (one declined to participate, and five had unilateral third molars). Consequently, 34 patients were enrolled in the study for an intention-to-treat (ITT) analysis. However, six patients were lost to follow-up as they did not return for the postoperative tomographic examination. Therefore, the final sample consisted of 28 patients for a treatment received (TR) analysis. This final sample included a total of 56 tooth extractions, involving 16 female and 12 male patients (Figure 3).

The participants’ ages ranged from 18 to 35 years (mean age of 22.36 ± 4.14 years). There were no statistically significant differences among the study variables in both the ITT and TR analyses. These variables included factors such as sample size, gender distribution, age, degree of impaction, surgical difficulty index, and procedure time. The absence of significant variation may reflect the careful standardization of the teeth and the consistent uniformity of the surgical procedures performed. Moreover it is worth noting that each patient served as his/her own reference and this may have contributed to this outcome (Table 1).

**Table 1:** Sample characterization.

Variable	TR analyses		ITT analyses	
	Control	Experimental	Control	Experimental
	n (%)	n (%)	n (%)	n (%)
Pernambuco index				
Low	19 (67.9%)	19 (67.9%)	24 (70.6%)	24 (70.6%)
Moderate	9 (32.1%)	9 (32.1%)	10 (29.4%)	10 (29.4%)
Pell & Gregory’s classification				
IA	5 (17.9%)	5 (17.9%)	6 (17.6%)	6 (17.6%)
IB	3 (10.7%)	3 (10.7%)	3 (8.8%)	3 (8.8%)
IIA	10 (35.7%)	10 (35.7%)	11 (32.4%)	11 (32.4%)
IIB	8 (28.6%)	8 (28.6%)	12 (35.3%)	12 (35.3%)
IIC	2 (7.1%)	2 (7.1%)	2 (5.9%)	2 (5.9%)
Winter’s classification				
Mesioangular	7 (25.0%)	7 (25.0%)	10 (29.4%)	10 (29.4%)
Vertical	19 (67.9%)	19 (67.9%)	22 (64.7%)	22 (64.7%)
Distoangular	1 (3.6%)	1 (3.6%)	1 (2.9%)	1 (2.9%)
Horizontal	1 (3.6%)	1 (3.6%)	1 (2.9%)	1 (2.9%)
Surgical time (min)*	8.07 ± 5.06	8.39 ± 6.94	7.26 ± 4.96	7.61 ± 6.57

*mean ^†^standard deviation; TR, treatment received; ITT, intention to treat; min, minutes.

### Pain and soft tissue assessments

The use of L-PRF in the alveoli resulted in a reduction in postoperative pain at both of the time intervals examined. A statistically significant difference in means was observed over the seven-day period (p = 0.019), while this difference was not apparent in the 12-hour follow-up (ITT analysis, p = 0.301; TR analysis, p = 0.307) (Table 2).

**Table 2:** Pain and probing depth assessment.

Variable	TR analyses				ITT analyses			
	Control	Experimental	p-value	Δ	Control	Experimental	p-value	Δ
VAS (cm)								
12h	3.36 ± 2.06	3.11 ± 1.85	0.301^a^	-0.25 ± 1.21	3.23 ± 2.06	3.00±1.79	0.307^a^	-0.23±1.18
7d	1.32 ± 1.81	0.64 ± 1.06	0.019^a^	-0.68 ± 1.39	1.12 ± 1.70	0.56±0.99	0.018^a^	-0.56±1.31
p-value	0.001^a^	<0.001^a^	-	-	0.001^a^	< 0.001^a^	-	-
DB (MM)								
PO	3.46 ± 0.64	3.46 ± 0.88	-	0.00 ± 0.77	-	-	-	-
3 mth	2.96 ± 0.69	2.89 ± 0.74	0.626^a^	-0.07 ± 0.77	-	-	-	-
p-value	0.003^a^	0.025^a^	-	-	-	-	-	-
D (mm)								
PO	3.66 ± 0.82	3.33 ± 0.51	-	-0.33 ± 1.03	-	-	-	-
3 mth	3.43 ± 0.74	3.14 ± 0.80	0.011^a^	-0.29 ± 0.53	-	-	-	-
p-value	1.000^a^	0.157^a^	-	-	-	-	-	-
DL (mm)								
PO	3.39 ± 0.63	3.50 ± 0.69	-	0.11 ± 0.74	-	-	-	-
3 mth	3.11 ± 0.63	3.04 ± 0.64	0.678^a^	-0.07 ± 0.90	-	-	-	-
p-value	0.087^a^	0.015^a^	-	-	-	-	-	-

*p < 0.05; Wilcoxon ^a^test. TR: treatment received; ITT: intention to treat; VAS, Visual Analog Scale; DB: distobuccal; D: distal; DL, distolingual; PO: preoperative assessment; mth = month.

### Periodontal condition

The assessment of the Healing Index (HI) and probing depth showed statistically significant differences at the one-month postoperative evaluation (p = 0.021) and the three-month follow-up for the distal (D) aspect (p =0.011), respectively (Tables 2 and 3). Furthermore, considering the probing depth at the distal sites of the second molar three months postoperatively, we observed no increase in these measurements from baseline to the 3-month postoperative follow-up time interval among any of the patients included in the trial. Consequently, based on this assessment, and within this context. we did not observe any instances of distal bone loss at the second molar

**Table 3:** Soft tissue healing and alveolar volume values.

Variable	Control	Experimental	p-value	Δ
	n (%)	n (%)		n (%)
Soft tissue healing scores (7 d)				
1	0 (0.0%)	0 (0.0%)	0.446	-
2	10 (35.7%)	5 (17.9%)		-
3	16 (57.1%)	19 (67.9%)		-
4	2 (7.1%)	4 (14.3%)		-
5	0 (0.0%)	0 (0.0%)		-
Soft tissue healing scores (1 mth)				
1	0 (0.0%)	0 (0.0%)	0.021	-
2	0 (0.0%)	0 (0.0%)		-
3	0 (0.0%)	0 (0.0%)		-
4	9 (32.1%)*	1 (3.6%)		-
5	19 (67.9%)	27 (96.4%)*		-
p-value	< 0.001	< 0.001		
Alveolar volume (PR 1)				
T0	622.86 ± 285.79mm^3^	590.10 ± 291.09mm^3^	0.127^a^	32.76 ± 181.25
T3	186.96 ± 131.97mm^3^	229.06 ± 146.09mm^3^	0.116^a^	-42.10 ± 150.55
p-value	<0.001^a^	<0.001^a^	-	
Variation (absolute number)	-435.90 ± 239.06mm^3^	-361.04 ± 224.76mm^3^	0.016^a^	-74.86 ± 184.19
Variation (%)	-68.96 ± 17.54mm^3^	-59.55 ± 20.62mm^3^	0.084^a^	-9.41 ± 22.54

*p < 0.05; McNemar’s test (n. %); ^a^Wilcoxon test; mth, month; PR 1: panoramic radiograph with a thickness of 1 mm.

### Bone repair imaging evaluation

In the volumetric analysis, there were no statistically significant differences observed between the control and experimental treatments at both T0 (p = 0.127) and T3 (p = 0.116) time intervals. However, both treatments showed a significant reduction in alveolar volume three months post-surgery (p < 0.01). Furthermore, the difference between T3 and T0 (Δ) exhibited a significant distinction (p = 0.016), with the control group demonstrating a more pronounced reduction in alveolar volume (Δ = -435.90 ± 239.06) compared with the experimental intervention group (Δ = -361.04 ± 224.76) (Table 3).

Relative to fractal analysis (FA), in the experimental intervention group, PR5 and SR1 values showed a consistent increase from T1 onwards. In contrast, in the control group, this increase became apparent only after T2. Nevertheless, it is important to note that the differences in trabecular complexity between the two treatments were not statistically significant during the one and three-month postoperative follow-up time intervals (p > 0.05) (Table 4).

**Table 4:** Fractal analysis for bone quality assessment.

Variable	Control	Experimental	p-value	Δ
PR 1				
T0	1.104 ± 0.075	1.097 ± 0.060	0.687^a^	-0.01 ± 0.09
T1	1.149 ± 0.070*	1.158 ± 0.054*	0.589^a^	0.01 ± 0.09
T3	1.068 ± 0.065*^†^	1.051 ± 0.059*^†^	0.225^a^	-0.02 ± 0.07
p-value	< 0.001^b^	< 0.001^b^	-	
PR 5				
T0	1.117 ± 0.066	1.114 ± 0.072	0.842^a^	0.00 ± 0.09
T1	1.142 ± 0.061	1.156 ± 0.060*	0.285^a^	0.01 ± 0.07
T3	1.077 ± 0.064*	1.067 ± 0.058*^†^	0.586^a^	-0.01 ± 0.09
p-value	< 0.001^b^	< 0.001^b^	-	
PR 10				
T0	1.126 ± 0.084	1.124 ± 0.064	0.881^a^	0.00 ± 0.08
T1	1.123 ± 0.079	1.144 ± 0.064	0.121^a^	0.02 ± 0.07
T3	1.103 ± 0.075	1.111 ± 0.057	0.666^a^	0.01 ± 0.10
p-value	0.248^b^	0.215^b^	-	
Mean PR				
T0	1.116 ± 0.059	1.112 ± 0.046	0.748^a^	0.00 ± 0.07
T1	1.138 ± 0.057*	1.152 ± 0.043*	0.139^a^	0.01 ± 0.05
T3	1.082 ± 0.052*^†^	1.077 ± 0.039*^†^	0.644^a^	-0.01 ± 0.06
p-value	0.001^b^	< 0.001^b^	-	
SR 1				
T0	1.165 ± 0.072	1.132 ± 0.066	0.056^a^	-0.03 ± 0.09
T1	1.195 ± 0.068	1.206 ± 0.037*	0.441^a^	0.01 ± 0.07
T3	1.103 ± 0.069*^†^	1.088 ± 0.068*^†^	0.335^a^	-0.01 ± 0.08
p-value	0.005^b^	< 0.001^b^	-	
Sagittal (internal)				
T0	1.106 ± 0.115	1.083 ± 0.114	0.350^a^	-0.02 ± 0.13
T1	1.123 ± 0.114	1.128 ± 0.075	0.796^a^	0.01 ± 0.12
T3	1.100 ± 0.114	1.108 ± 0.075	0.712^a^	0.01 ± 0.11
p-value	0.105^b^	0.409^b^	-	

^a^Wilcoxon test; ^b^Friedman/Dunn test. *p < 0.05 vs. T0; ^†^p < 0.05 vs. T1; PR 1: panoramic radiograph with a thickness of 1 mm; PR 5: panoramic radiograph with a thickness of 5 mm; PR 10: panoramic radiograph with a thickness of 10 mm.

## Discussion

In the present split-mouth study, in which each patient served as both the experimental and control individuals, no significant differences were observed between the treatments in terms of degree of impaction and procedure time. These findings suggested a potential reduction in bias, thereby enhancing the precision of assessing the influence of L-PRF on alveolar healing after mandibular third molar surgery.

Despite the extensive body of existent(?) research, a lack of appropriate standardization and methodological design has been observed, contributing to conflicting outcomes and controversies within the literature.^16^ Consequently, this investigation adhered to a methodological framework similar to that of studies conducted by Ritto et al.,^9^ Kapse et al.,^17^ and Ozgul et al.^18^ These studies, classified in a systematic review with meta-analysis by Zhu et al.^19^ as exhibiting a low risk of methodological bias, were used to guide the methodology of this study.

One of the most extensively scrutinized clinical parameters is pain, a highly subjective experience that varies from patient to patient. This underscores the significance of using a split-mouth clinical trial design. Whereas some researchers opt to use the number of analgesics consumed as an indicator of pain intensity,^20^ the present study focused solely on the Visual Analog Scale (VAS) as the assessment tool. This choice was motivated because the VAS is the measurement most widely used and frequently referenced in the literature.^9,17,18^

Relative to the timing of pain analysis, we conducted evaluations at two specific time intervals. First, a 12-hour post-surgery assessment was performed, as this time interval typically represents the peak of pain. In addition, a follow-up assessment occurred one week after surgery, as this time interval is commonly used to conclude postoperative evaluations for surgical procedures. We observed an improvement in pain levels among patients who received L-PRF at both evaluation time intervals. However, statistically significant improvement was only evident during the one-week follow-up. One possible explanation for this outcome could be that since tooth extractions occurred on the same day, patients may have encountered challenges when distinguishing and individually evaluating each surgical site immediately after the procedure. This perspective aligns with the findings presented by Ozgul et al.,^18^ which could be considered a limitation of our study.

When evaluating split-mouth studies, variations in outcomes have appeared Some investigations have pointed out the potential of L-PRF to mitigate postoperative pain.^17^ Conversely, other studies have proposed that L-PRF might not significantly influence this outcome,^9,19^ raising questions about its effectiveness. Similarly, while certain authors have reported that L-PRP could enhance soft tissue healing, others have found no significant distinctions.^9,17^ In the present study, patients exhibited notable enhancements in both pain severity and soft tissue healing.

The periodontal condition of the distal surface of the second molar may have had a direct influence on the healing process in the region adjacent to and near the third molar. The application of L-PRF to fill the alveolus could promote effective healing by potentially reducing the infiltration of microorganisms, thus positively affecting the periodontal health of the mandibular second molar. This, in turn, would reduce the likelihood of periodontal pocket formation, resulting in a decrease in probing depth, as demonstrated in our study.

Analysis of prior research may contribute to understanding the mechanisms underlying the benefits of L-PRF in pain alleviation, by improving soft tissue healing, and improving periodontal diseases.

In post-extraction sockets, L-PRF has been demonstrated to enhance epithelialization and reduce postoperative discomfort, as demonstrated by higher levels of healing and decreased levels of pain and analgesic consumption reported in L-PRF-treated patients in comparison with controls.^21^ This suggested that L-PRF may exert its effects by promoting tissue regeneration and modulation of pain perception.

According to a study evaluating the impact of L-PRF on soft tissue healing and its relationship with local growth factor and cytokine concentrations, tissue healing scores were higher, and there was less postoperative pain at L-PRF sites. This was correlated with increased levels of growth factors such as platelet-derived growth factors (PDGFs) and basic fibroblast growth factor (bFGF), which are known to play roles in tissue repair and regeneration.^22^

The regenerative potential of L-PRF in periodontal therapy is related to its capacity to enhance both hard and soft tissue regeneration. A fibrin network formed by the gradual polymerization that occurs during PRF preparation promotes effective cell migration and proliferation, which enhances cicatrization.^9^ The therapeutic benefits of L-PRF in treating periodontal hard and soft tissue abnormalities are probably due in part to this mechanism. Moreover, it was observed that the use of L-PRF increased bone density after extraction of the mandibular third tooth, suggesting that it had a favorable impact on bone healing.^23^ This might be because the L-PRF matrix releases growth factors that could promote osteogenesis.

In terms of complications, only one single case of alveolar osteitis and one instance of postsurgical bleeding were observed, both occurring on the control side. Alveolar osteitis, though relatively infrequent, can generate significant discomfort for patients after the procedure. According to Zhu et al.,^19^ while L-PRF may not entirely prevent this complication, it might contribute to reducing its incidence. Relative to bleeding, the use of L-PRF tends to lower the risk of post-surgical bleeding due to the platelet aggregation facilitated by the higher platelet concentration.^17^

This trial focused on L-PRF and its impact on clinical and imaging outcomes after third molar surgery. Conversely, some evidence has been shown to suggest that different types of PRF can have varying effects on pain and healing outcomes after third molar surgeries. A study comparing advanced PRF (A-PRF) and standard PRF (S-PRF) found that A-PRF resulted in significantly improved pain, swelling, and mouth opening outcomes in comparison with S-PRF.^24^ This suggested that there may be differences between types of PRF in terms of their efficacy. Thus, the extent of these benefits and the differences between types of PRF require further investigation to fully understand their clinical implications.

In terms of cost-benefit, while the studies did not provide explicit economic analyses, the use of L-PRF is derived from the patient’s blood, which may reduce the costs associated with synthetic or donor materials and the risk of disease transmission. Furthermore, the simplicity of the technique suggested that it may not require significant additional resources or specialized equipment, potentially offering a cost-effective treatment option.^25^ Despite these benefits, we believe that this intervention should not be generalized. It is important to thoroughly assess each patient’s medical history, overall health status, and specific surgical needs to determine whether L-PRF therapy would be appropriate for them. In the majority of cases, L-PRF can be a valuable adjunctive treatment in promoting wound healing, reducing inflammation, and enhancing tissue regeneration in oral surgical procedures as observed in the present trial. However, there is a lack of evidence-based practice regarding the use of L-PRF in patients with blood disorders.

## Conclusion

In summary, the finding os this clinical trial’s emphasize the positive impact of L-PRF on various postoperative clinical parameters, including pain, soft tissue healing, and periodontal condition. These results suggest that L-PRF has the potential to enhance alveolar ridge preservation and expedite the initial healing process after mandibular third molar extraction. This means that L-PRF may be a useful alternative, with a cost-benefit ratio, in extraction cases in which there is a higher risk of postoperative morbidity and in other areas where implant rehabilitation is required in the future. The present study maintains strong internal validity due to its rigorous methodology, although its external validity is somewhat limited by specific sample characteristics. In view of the critical importance of this topic in the field of oral sciences, there is a compelling need for future well-designed trials to investigate the use of L-PRF in third molar surgeries across diverse populations. This type of research should prioritize patient-centered outcomes to provide more comprehensive insights into the clinical advantages of L-PRF.
